# EXPLORING THE NEIGHBORHOOD ENVIRONMENT’S ROLE IN OUTDOOR WALKING EXPERIENCES OF PEOPLE LIVING WITH DEMENTIA

**DOI:** 10.1093/geroni/igae098.1333

**Published:** 2024-12-31

**Authors:** Habib Chaudhury, Kishore Seetharaman, Joey Wong, Lillian Hung, Cari Randa-Beaulieu, Shannon Freeman, Mark Groulx

**Affiliations:** Simon Fraser University, Vancouver, British Columbia, Canada; Simon Fraser University, Vancouver, British Columbia, Canada; University of British Columbia, Vancouver, British Columbia, Canada; University of British Columbia, Vancouver, British Columbia, Canada; Alzheimer Society of British Columbia, Vancouver, British Columbia, Canada; University of Northern British Columbia, Prince George, British Columbia, Canada; University of Northern British Columbia, Prince George, British Columbia, Canada

## Abstract

This presentation is based on a study that explores how people with dementia experience their neighborhood environment in the context of everyday outdoor walking. The experientially grounded approach explored ways in which people living with dementia can inform the process of dementia-friendly and inclusive planning and design. The focus on lived experience can also help planners and designers gain insights into the actions and strategies that people living with dementia adopt to cope with challenges in mobility and navigation in the neighborhood, thus gaining useful knowledge to generate contextually relevant design solutions. Four-part sequential sit-down and video-documented walk-along interviews were conducted with 32 participants living with mild to moderate dementia or mild cognitive impairment in British Columbia, Canada. Thematic analysis of interview transcripts and videos helped identify themes relevant to the ways in which the neighborhood environment shaped participants’ outdoor walking experiences. Findings underscored both supportive and constraining aspects of the neighborhood environment as it pertains to outdoor walking. Awareness of environmental barriers and stressors in the context of their physical and cognitive challenges informed participants’ planning and decision-making processes in relation to outdoor walks, as well as coping strategies to enhance familiarity, safety, comfort, and independence in the neighborhood environment. Planning and designing supportive built environments is key to facilitating the outdoor mobility of people living with dementia and should be a focus of research, policy, and practice related to building dementia-inclusive communities.

